# Telephone-Based Health Coaching: Reduction in Alzheimer’s Risk Behaviors

**DOI:** 10.1093/geroni/igaa057.890

**Published:** 2020-12-16

**Authors:** Annie Rhodes, Taylor Wilkerson, Jennifer Inker, Joann Richardson, Faika Zanjani

**Affiliations:** 1 Virginia Commonwealth University, Richmond, Virginia, United States; 2 VCU, School of Social Work, Richmond, Virginia, United States; 3 VCU, Richmond, Virginia, United States

## Abstract

Objective: Explore the feasibility of integrating intensive, telephone-based health coaching programs in low-income senior housing communities to reduce Alzheimer’s risk behaviors. Design: Participants meeting study criteria: 60 years or older, a working telephone, no cognitive diagnoses, income below 1,000 USD monthly, and active cardiovascular or diabetic health symptoms were recruited from low-income housing units. Engagement in Alzheimer’s risk behaviors: Cigarette use, alcohol overuse, polypharmacy, inactivity, depression, and cognition status, were measured at enrollment, and 12 weeks post. Weekly coaching sessions focused on reducing behavioral risk for Alzheimer’s disease. Setting: Low-income senior apartments in Richmond, Virginia Participants: Twenty older adults, living in low income senior high rises. Participants were majority (95%) African-American (Mean= 69 years, SD=4.17, Range=61-77). Intervention: Participants engaged in a call with a coach for 12 weeks, focused on Alzheimer’s risk reduction. Participants identified with coach specific behaviors to target. Primary Outcome Measure: Feasibility of telephone-based health coaching to reduce Alzheimer’s Risk Behaviors. Feasibility is defined as participant engagement in health coaching and self-rated health outcomes. Results: Of the original 20 enrollees, 19 (95%) participated in coaching sessions. On average, 8.75 sessions were completed. All participants rated their experience as positive, and self-reported an improvement in health and healthy behaviors, in exit interviews. The coaching experience was rated 94.11 on a scale from (0-100). Participants rated their health coach, on average, 90.44 on a scale from (0-100). Participants rated their health improved as 92.37 on a scale from (0-100). Conclusion: Telephone-based health coaching was feasible based on participant engagement.

